# Autism Genetics: Over 100 Risk Genes and Counting

**DOI:** 10.15844/pedneurbriefs-34-13

**Published:** 2020-12-04

**Authors:** Marc P. Forrest, Peter Penzes

**Affiliations:** 1 Department of Physiology, Northwestern University, Chicago, IL; 2 Department of Psychiatry and Behavioral Sciences, Northwestern University, Chicago, IL; 3 Center for Autism and Neurodevelopment, Northwestern University, Chicago, IL

**Keywords:** Autism Spectrum Disorder, Genetics, Exome Sequencing

## Abstract

Researchers from the Autism Sequencing Consortium (ASC) led by Joseph Buxbaum at the Icahn School of Medicine at Mount Sinai report the largest exome sequencing study in autism spectrum disorder (ASD) to date.

Researchers from the Autism Sequencing Consortium (ASC) led by Joseph Buxbaum at the Icahn School of Medicine at Mount Sinai report the largest exome sequencing study in autism spectrum disorder (ASD) to date. Combining sequencing data from 35,584 individuals, including 11,986 with ASD, they implicate a total of 102 genes in disease risk. By analyzing de novo variants in ASD probands, the authors identify a 3.5-fold enrichment of protein-truncating variants in loss-of-function intolerant genes and a 2.1-fold enrichment of damaging missense variants in ASD cases. Four risk genes (*SLC6A1*, *DEAF1*, *KCNQ3*, *SCN1A*) were associated with a disproportionate enrichment in missense variants compared to protein-truncating variants, suggesting that gain-of-function mechanisms may increase liability for ASD in this subset. Of the 102 risk genes, 12 genes were located in regions impacted by copy number variation (CNV), potentially nominating driver genes in CNVs associated with ASD.

To isolate genes with a bias for ASD without severe neurodevelopmental co-morbidities, the authors compared the rate of disruptive de novo variants identified in ASD to rates of de novo variants identified in severe neurodevelopmental disorders (NDDs). This analysis yielded 53 risk genes with a bias for ASD and 49 genes with a bias for NDDs. Individuals with variants in NDD-predominant risk genes were associated with later age of walking and a lower full-scale IQ than individuals with variants in ASD-predominant risk genes. Functionally, the 102 risk genes clustered into three main categories, including gene expression regulation (58), neuronal communication (24), and cytoskeletal organization (9). The authors conclude that the ASD phenotype arises from diverse neurobiological mechanisms, and dissecting the convergence points of these pathways will be central to understanding the disorder. [1]

COMMENTARY. This article is a landmark study providing a comprehensive analysis of autism genetics and revealing essential genes, cell-types, and time points most associated with the disease. Although most of the genetic risk in ASD is due to common variation, only a few risk polymorphisms have been identified [2]. However, exome sequencing studies of rare variants such as this study have provided critical insight into ASD etiology. Despite rare variants cumulatively representing ~5% of individuals, these variants often significantly affect disease risk and represent a fundamental entry point for disease modeling and mechanistic understanding of disease [3,4].

Interestingly this study implicates both excitatory and inhibitory neurons in ASD pathogenesis. In concert with data from animal and cellular models, these data support the notion that dysfunctional excitation or inhibition may lead to ASD via an imbalance of brain circuitry [5]. Given the functions of the 102 risk genes, these neurobiological deficits may be most commonly caused by directly impacting neuronal communication (e.g., synapses) or by dysregulating gene expression during brain development.

## Disclosures

The authors have declared that no competing interests exist.
